# Peripheral airways involvement in children with asthma exacerbation

**DOI:** 10.1111/crj.13456

**Published:** 2021-10-29

**Authors:** Maria Wawszczak, Marek Kulus, Joanna Peradzyńska

**Affiliations:** ^1^ Department of Pediatric Pneumonology and Allergy Medical University of Warsaw Warsaw Poland; ^2^ Department of Epidemiology and Biostatistics Medical University of Warsaw Warsaw Poland

**Keywords:** acute asthma, impulse oscillometry, inert gas washout, small airways disease

## Abstract

**Objective:**

The literature provides some evidence of peripheral airways key role in the pathogenesis of asthma. However, the extent to which lung periphery including acinar zone contribute to asthma activity and control in pediatric population is unclear. Therefore, the aim of the study was to estimate peripheral airways involvement in children with asthma exacerbation and stable asthma simultaneously via different pulmonary function tests.

**Methods:**

Children with asthma exacerbation (*n* = 20) and stable asthma (*n* = 22) performed spirometry, body plethysmography, exhaled nitric oxide, impulse oscillometry (IOS), and multiple‐breath washout (MBW).

**Results:**

Peripheral airway's function indexes were increased in children with asthma, particularly in group with asthma exacerbation when compared with stable asthma group. The prevalence of abnormal results was significantly higher in asthma exacerbation. All children with asthma exacerbation had conductive ventilation inhomogeneity; 76% had acinar ventilation inhomogeneity. According to IOS measurements, resistance and reactance were within normal range, but other IOS parameters were significantly higher in children with asthma exacerbation compared with stable asthma group. The 36% of children with acute asthma had air trapping.

**Conclusion:**

Significant involvement of peripheral airways was observed in children with asthma, particularly in asthma exacerbation, which determine lung periphery as important additional target for therapy and provide new insights into pathophysiological process of pediatric asthma.

## 1 INTRODUCTION

Asthma is a heterogeneous condition characterized by airway hyperresponsiveness and variable airflow limitation in response to diverse of stimuli.^1^ Historically, asthma was understood primarily the disease of large and medium airways, while the peripheral airways were considered almost insignificant for its development and control.^2^ Nonetheless, over the past few years, there has been evidence of the peripheral airways involvement in the course of asthma, regardless of its severity.^3^, ^4^ Moreover, they can be the main site of airflow limitation in patients with asthma.^5^ Clinical studies suggested significant relevance of peripheral airways inflammation to asthma morbidity indicating their relationship to asthma severity^6^, ^7^, ^8^, ^9^ or control^10^, ^11^, ^12^, ^13^ and demonstrating their involvement in acute asthma.^14^, ^15^ To date, studies utilizing pulmonary function tests in assessment of pediatric asthma shown involvement predominantly of the conductive peripheral airways, but acinar zone seems to be mostly insignificant in pathological process.^16^ However, the disturbance within this area of the lung were noted in some children with unstable asthma.^10^ Moreover, pathomorphological studies in acute fatal asthma confirmed significant involvement of the peripheral airways including acinar spaces in pathological process.^17^, ^18^ The results have shown extensive inflammatory, as well as structural changes throughout whole lung periphery. Given the above findings, the authors hypothesized that peripheral airways including acinar zone may play an important role in asthma activity in children. The evaluation of the impact of peripheral airways on asthma instability/activity may be crucial to achieve control and reduce risk of asthma‐related death. It is worth mentioning that small airways are less accessible to inhaled medication and a different therapeutic approach, including extra‐fine particle inhaled corticosteroids (ICS) or systemic steroid treatment, is required to treat pathology that is predominantly situated in this area of the lung.^19^

Detection and quantification pathology within lung periphery are available with pulmonary function tests that reflect premature airway closure, airway resistance, air trapping, and regional airflow heterogeneity.^2^ One of those methods is body plethysmography, which measures the functional residual capacity (FRC), residual volume (RV), and its ratio to total lung capacity (RV/TLC) possibly indicating air trapping, when elevated.^20^ The others include novel methods, nitrogen multiple‐breath washout (N2MBW) test, quantifying the ventilation inhomogeneity, and impulse oscillometry (IOS), measuring airways resistance and elastic properties of lung periphery.^21^, ^22^ Each of the above is examining different aspects of peripheral airways pathology, and their combination can provide complementary information on peripheral lung function. To the date, only a few studies have demonstrated utility of simultaneously performed different pulmonary function tests in both childhood and adult asthma, but it appears that only their combination can provide complementary information on peripheral lung function.^23^, ^24^, ^25^, ^26^ Moreover, the important aspects of distal airways function could be missed if only one physiological variable is used for the assessment.^26^

Therefore, the primary aim of the study was to estimate peripheral airways involvement in children with asthma exacerbation and stable asthma assessed simultaneously via various pulmonary function measurements.

## 2 METHODS

The study was approved by the Ethics Committee of the Medical University of Warsaw.

Patients of the Department of Pediatric Pneumonology and Allergy, Medical University of Warsaw, with stable asthma and asthma exacerbation were enrolled into the study between November 2018 and October 2019. The stable asthmatic group was recruited from outpatient clinic, and the acute asthmatic group from patients admitted to the hospital because of exacerbation. The study involved only children who were able to perform lung function tests, whose parents/caregivers signed informed consent for participation.

The asthma diagnosis and severity assessment were based on Global Initiative for Asthma (GINA) guidelines.^1^ The current asthma control was determined using the Asthma Control Test (ACT) and, for children below 12 years of age, the childhood Asthma Control Test (cACT).^27^, ^28^

The exclusion criteria included diagnosis of other chronic pulmonary disease, congenital lung malformation, history of prematurity, low birth weight, need for mechanical ventilation in neonatal period, or diagnosis of bronchopulmonary dysplasia. In those who underwent chest radiography, presence of consolidation or hilar adenopathy was considered as a reason for exclusion.

Children were assigned to the stable asthma group if they had reported no exacerbations or changes in symptoms/medication use for at least 6 weeks before visit and they had ACT or cACT score of more than 20.

Asthma exacerbation was defined as an acute or subacute episode of progressive worsening in symptoms (shortness of breath, cough, chest tightness, tachypnea, and wheezing).^1^

### 2.1 Procedures

All patients underwent pulmonary function tests including spirometry, body plethysmography, IOS, N2MBW test, and fractional exhaled nitric oxide (FeNO) measurement. All procedures were performed in agreement with the American Thoracic Society/European Respiratory Society (ATS/ERS) recommendations.^29^, ^30^

Spirometry and IOS were performed using the Vyntus IOS (CareFusion, San Diego, CA, USA), body plethysmography using the Master Screen Body (CareFusion), N2MBW using the Exhalyzer®DN2 MBW device (Eco Medics AG, Duernten, Switzerland), and FeNO with NOA 280i (Sievers; GE Analytical Instruments, Boulder, CO, USA).

The following parameters were recorded:

Spirometry: forced expiratory volume in 1 s (FEV1), forced vital capacity (FVC), and forced expiratory volume in 1 s to forced vital capacity ratio (FEV1/FVC ratio).

Body plethysmography: specific airway resistance (sReff), total lung capacity (TLC), RV, and RV/TLC ratio.

IOS: resistance at 5 Hz (R5) and resistance at 20 Hz (R20), frequency dependence of resistance (R5–R20), reactance at 5 Hz (X5), area under the reactance curve (AX), and resonance frequency (Fres).

N2MBW: lung clearance index (LCI) at 2.5% of the initial concentration of N2, conductive ventilation inhomogeneity (Scond), and acinar ventilation inhomogeneity (Sacin).

All measured parameters were presented as *z*‐scores. The exceptions were FeNO, R5–R20, and AX, which were presented only as absolute values due to lack of the reference equations. Global Lung Function Initiative 2012 and Dencker and Berdel/Lechtenboerger reference values were used. For N2MBW, values provided by the manufacturer were applied. The abnormal results were defined as *z*‐score values outside of −1.65 to 1.65.

### 2.2 Statistics

Statistical analysis was performed with the Statistica 12 software package (StatSoft, Inc., Tulsa). Data are presented as median and interquartile range.

Because several lung function variables did not show normal distribution, non‐parametric tests were used. Comparison between groups and subgroups were performed using Mann–Whitney's *U*‐tests for interval variables and *χ* ^2^ test was used to compare proportions. Possible relationships between different variables were evaluated using Spearman's rank correlation test. The two‐tailed significance level was set at *p* < 0.05.

## 3 RESULTS

### 3.1 Baseline patient characteristics

Forty‐two children divided into two subgroups (20 with asthma exacerbation and 22 with stable asthma) aged 6–17 years (median 12.5 years) were included. Patients' characteristics are shown in Table 1. There were no statistical differences in terms of age, height, weight, and body mass index (BMI) between two study groups. The groups were also comparable in terms of asthma severity on GINA score. Only the median ACT score was significantly lower in patients with asthma exacerbation. Most of the patients with asthma were on regular treatment.

**TABLE 1:** Patient's characteristics

	Stable asthma	Asthma exacerbation
	*n* = 22	*n* = 20
Age (years)^*^ ^,^ ^a^	12 (7–17)	12.5 (6–17)
Male sex/whole group^*^ ^,^ ^b^	16/22	13/20
Height (cm)^*^ ^,^ ^a^	155.5 (143–171)	157.0 (140.5–163.5)
Weight (kg)^*^ ^,^ ^a^	47.5 (39–59)	51.5 (36–69)
BMI (kg/m^2^)^*^ ^,^ ^a^	19.5 (18–23)	21.5 (16.5–25)
ACT score^**^ ^,^ ^a^	23 (23–25)	19 (17–20)
FeNO (ppb)^*^ ^,^ ^a^	30.5 (11–54)	33.5 (26–54)
Medication use		
Inhaled corticosteroid^*^ ^,^ ^b^	77% (17/22)	90% (18/20)
Inhaled corticosteroid and long‐acting β agonist^*^ ^,^ ^b^	50% (11/22)	40% (8/20)
Inhaled corticosteroid and leukotriene antagonist^*^ ^,^ ^b^	9% (2/22)	5% (1/20)
Asthma severity		
GINA 1–2^*^ ^,^ ^b^	41% (9/22)	40% (9/20)
GINA 3^*^ ^,^ ^b^	32% (7/22)	40% (5/20)
GINA 4–5^*^ ^,^ ^b^	27% (6/22)	30% (6/20)

*Note*: Data are presented as median and interquartile range.

Abbreviations: ACT, Asthma Control Test; BMI, body mass index; FeNO, fractional exhaled nitric oxide; GINA, Global Initiative for Asthma.

^a^ Mann–Whitney *U*‐test.

^b^ *χ* ^2^ test.

* *p* > 0.05.

** *p* < 0.05.

### 3.2 Comparison of pulmonary function parameters

In asthma exacerbation group, 70% of FEV1 were abnormal and in stable asthma group, the percentages were 9% (Table 2). There were 36.4% versus 85% patients with obstructive ventilatory defect (defined as FEV1/FVC ratio < −1.65 of *z*‐score) in stable versus exacerbation asthma group.

**TABLE 2:** Pulmonary function measurements and observed differences between study groups

Parameter	Stable asthma (*n* = 22)	Asthma exacerbation (*n* = 20)
FEV1**	0.0 (−1.37 to 0.59)	−1.98 (−3.01 to 1.42)
FVC**	0.46 (−0.07 to 1.29)	−0.56 (−2.7 to 0.67)
FEV1/FVC %**	−1.35 (−1.76 to −0.36)	−2.15 (−2.46 to −1.83)
RV*	2.53 (0.67 to 3.5)	−0.46 (−1.04 to 2.23)
TLC*	0.11 (−0.64 to 1.01)	−0.66 (−2.11 to 1.36)
RV/TLC %*	−0.60 (−1.66 to −0.11)	−0.11 (−1.32 to 4.09)
sReff**	2.53 (0.67 to 3.5)	5.06 (0.3 to 16.9)
R5*	−0.05 (−0.76 to 0.32)	−0.04 (−0.43 to 0.61)
R20*	−0.21 (−0.73 to 0.32)	−0.31 (−0.98 to 0.11)
R5–R20 (kPa/L/s)**	0.12 (0.07 to 0.16)	0.18 (0.13 to 0.295)
X5*	0.2 (−0.17 to 0.63)	−0.23 (−0.8 to 0.29)
AX (kPa/L)**	0.75 (0.44 to 1.33)	1.4 (1.20 to 2.65)
Fres**	0.69 (−0.14 to 1.36)	1.37 (0.955 to 1.985)
Scond**	0.35 (−0.5 to 1.5)	7.7 (4.6 to 8.9)
Sacin**	0.4 (−1.3 to 1.7)	2.2 (1.6 to 3.2)
LCI**	1.6 (0.7 to 3.4)	8.2 (5.85 to 11.4)

*Note*: All results are presented as *z*‐scores, except R5–R20 and AX. Data are presented as median and interquartile range. All variables compared by Mann–Whitney *U*‐test.

Abbreviations: AX, area under the reactance curve; FEV1, forced expiratory volume in 1 s; Fres, resonant frequency; FVC, forced vital capacity; LCI, lung clearance index; R20, resistance at 20 Hz; R5, resistance at 5 Hz; R5–R20, frequency dependence of resistance; RV, residual volume; RV/TLC, ratio of residual volume to total lung capacity; Sacin, acinar ventilation inhomogeneity; Scond, conductive ventilation inhomogeneity; sReff, specific airway resistance; TLC, total lung capacity; X5, reactance at 5 Hz.

* *p* > 0.05.

** *p* < 0.05.

Body plethysmography showed significantly higher specific resistance in asthma exacerbation group (Table 2). The abnormally increased RV/TLC ratio, showing air trapping, was noted only in children with asthma exacerbation (in 36.8% of patients).

There were also significantly increased some IOS variables reflecting peripheral airways function, that is, R5–R20, Fres, and AX (Table 2). The median of AX and R5–R20 values, as well as median *z*‐scores of Fres were significantly higher in asthma exacerbation group when compared with stable asthma group. However, the median *z*‐score of Fres, as well as median *z*‐scores of X5 and R5 were within normal range either in children with stable asthma or asthma exacerbation.

All MBW parameters, that is, LCI, Scond, and Sacin, were abnormally increased in asthma exacerbation group (Table 2). The median *z*‐scores of all MBW parameters in stable asthma group were within normal range. However, the median *z*‐score of LCI was near upper limit of normal due to increased values in patients with moderately severe disease. There were also noted independently abnormal values of Scond and Sacin (22.7% and 27%, respectively) in patients with stable asthma. In contrast, all patients with asthma exacerbation had abnormal Scond values and vast majority (76%) of patients had abnormal Sacin values.

The median of exhaled NO values in children with asthma exacerbation was higher than in children with stable asthma, but the difference did not reach statistical significance.

### 3.3 Association between standard lung function tests and IOS, MBW measurements

To see possible relationship between standard lung function tests (i.e., spirometry and body plethysmography) and those reflecting peripheral airway function (IOS and N2MBW), various parameters were correlated. Detailed information of the correlations found between lung function parameters are shown in Table 3.

**TABLE 3:** Association between standard lung function tests and IOS, MBW measurements

	R5	X5	Fres	AX	R5–R20	LCI	Scond	Sacin
FEV1	*	*	−0.6**	−0.62**	−0.66**	−0.62**	−0.55**	*
sReff	0.44**	−0.3**	0.33**	0.45**	0.4**	0.54**	0.45**	*
RV/TLC	*	*	*	0.49**	*	0.38**	0.34**	*

*Note*: Data are presented as *r*‐Spearman value; all except AX and R5–R20 were calculated for *z*‐scores.

Abbreviations: AX, area under the reactance curve; FEV1, forced expiratory volume in 1 s; Fres, resonant frequency; LCI, lung clearance index; R20, resistance at 20 Hz; R5, resistance at 5 Hz; R5–R20, frequency dependence of resistance; RV, residual volume; RV/TLC, ratio of residual volume to total lung capacity; Sacin, acinar ventilation inhomogeneity; Scond, conductive ventilation inhomogeneity; sReff, specific airway resistance; X5, reactance at 5 Hz.

* *p* > 0.05.

** *p* < 0.05.

### 3.4 Association between IOS and MBW measurements

There was a weak positive correlation of LCI with R5–R20 (*r*‐Spearman 0.39, *p* = 0.01) and with AX (*r*‐Spearman 0.35, *p* = 0.02) and Scond with AX (*r*‐Spearman 0.34, *p* = 0.03) in whole study group.

### 3.5 Association of IOS, LCI measurements with GINA scores

Positive correlation between GINA scores and LCI was confirmed (*r*‐Spearman 0.36, *p* = 0.02). Any associations with other N2MBW parameters and IOS measurements were not confirmed.

## 4 DISCUSSION

The results of this study confirmed substantial involvement of peripheral airways in pediatric asthma. Most of the parameters reflecting peripheral airway's function were significantly increased in children with asthma exacerbation, that is, R5–R20, AX, Fres, LCI, Scond, and Sacin. Also, specific airway resistance measured in body plethysmography was remarkably increased in asthma exacerbation, and more than one third of patients from this group presented air trapping. Some involvement of the peripheral airways was observed either in children with stable asthma, but less significant when compared with asthma exacerbation group.

The study detected presence of excessive ventilation inhomogeneity within conductive and acinar zone in children with asthma exacerbation expressed by the abnormally increased Scond and Sacin values. These results are different than those found in previous studies on pediatric asthma presenting subtle changes in all N2MBW parameters but with the predominance only of increased Scond values.^16^ The acinar ventilation heterogeneity detected by MBW test was rarely found in children with asthma, even in a case of moderately severe disease and was reported predominantly in study evaluating children with unstable asthma.^9^, ^10^ Keen et al. in their study found additionally an association of Sacin with exhaled alveolar NO concentration, what might suggest that Sacin reflects inflammation process within the most peripherally located airways.^10^ Basing on the above findings and this study outcomes, it can be hypothesized that N2MBW might offer information on the disease activity and Sacin may reflect the very pronounced inflammation process reaching the acinar region, which occurs mostly in asthma exacerbation. This pointed that area of the lung as a possible additional target for asthma therapy. However, it needs more evidence and requires further investigations.

For evaluation mechanical properties of the peripheral airways, the IOS was performed. The results shown significantly increased R5–R20, AX, and Fres values in children with asthma exacerbation when compared with stable asthma group. In contrast, the commonly assessed IOS measurements, such as X5 and R5, remained within normal range, either in children with asthma exacerbation and stable asthma. That observation may suggest that parameters such as resistance and reactance evaluated in IOS are not particularly useful, but novel parameters, such as resonant frequency, area under the curve, and difference in R20–R5, are more sensitive in clinical practice. This is in accordance with other studies results concerning sensitivity of conventional IOS parameters, X5 and R5, in detection functional abnormalities in lung periphery in young children.^31^ Knihtilä et al. showed the inability of X5 and R5 *z*‐scores to distinguish children with current or past lower respiratory tract symptoms from healthy children.^31^ The authors also indicated the superiority of AX and R5–R20 over R5 and X5 in evaluation of pediatric asthma. Additionally, regarding the use of reference values, Tirakitsoontorn et al. found that proposed in their study selective cut‐off points of IOS measures, based on the clinical outcome of unstable asthma, could fall within normal values and still be clinically meaningful.^32^

To assess the impact of peripheral airways on airflow limitation in children with asthma, the relationship between FEV1 and peripheral airways parameters was reviewed. The study showed correlation of FEV1 with Scond and AX, R5–R20, which may suggest that the function of peripheral airways is an important determinant of airflow limitation in children with asthma. The study didn't revealed association between FEV1 and Sacin neither in children with asthma exacerbation nor stable asthma, what is inconsistent to findings in acute and severe adult asthma presented by Thompson et al., showing strong correlation of FEV1 with Sacin.^15^ The authors speculated that acinar ventilation heterogeneity is a major contributor to airflow obstruction in patient with unstable asthma.

The recent evidence from the study by Mahut et al. and Sorkness et al. indicated air trapping as the most profound stage of peripheral airway narrowing and significant prognostic factor of asthma severity and instability.^33^, ^34^ In the present study, air trapping was present only among some children with asthma exacerbation; it was in just over a third of patients. On the other hand, abnormal results of remaining tests reflecting the functional status of the peripheral airways were observed much more frequently, that is, mentioned above IOS parameters and N2MBW parameters. That is, inflammation in this area does not always have to lead to complete airway closure. The study results showed an association of RV/TLC ratio with AX, but not with R5–R20, found in asthma exacerbation group, suggesting that area of the reactance better reflects the function of narrowed peripheral airways. This observation is in accordance with previous report by Goldman et al. showing significant increase of airway reactance at low frequencies in hyperinflated lungs, in contrast to airway resistance, which has been mostly normal.^35^ In the study by Downie et al., including adults, the correlation between X5 and Sacin, and air trapping in patients with methacholine‐induced bronchoconstriction were noted.^25^ What was interesting in this study, accordingly to N2MBW and IOS measurements, RV/TLC ratio was related to Scond, AX, but not to Sacin or X5. This observation pointed some discrepancies between peripheral airways assessment in pediatric and adult asthma.

Patients with obstructive ventilatory defect usually show increased airway resistance on body plethysmography. However, sReff is not thought to specifically reflect distal airway dysfunction.^2^ Perhaps it is time to change this common view? The study results showed positive correlation of IOS and N2MBW parameters with specific airways resistance derived from body plethysmography. What may indicate that narrowing of peripheral airways is an important compound of increased airway resistance of the whole bronchial tree, particularly in asthma exacerbation. These findings are consistent with recent studies outcomes.^36^, ^37^ Yanai et al. using wedged bronchoscopy found that peripheral airways resistance accounted for 24% of total airway resistance in healthy adults, rising to 34% in asymptomatic newly diagnosed patients with asthma and as high as 51% in patients with severe asthma.^36^ Moreover, Wagner et al. demonstrated that the peripheral airways resistance in asymptomatic asthma patients with normal spirometry and normal plethysmographic airway resistance has been more than sevenfold higher than in healthy subjects.^37^

Although most of the parameters measured by N2MBW and IOS reflect abnormal ventilation in the peripheral airways, they may show different aspects of airway pathology. When seeking for correlations between N2MBW measurements and IOS variables, it occurred that only few were detected, which is in concordance to previous reports.^23^, ^24^, ^25^, ^26^ The study showed that higher values of Scond determined higher AX. Interestingly, such relationship between Scond and R5–R20 was not observed. This could be explained that R5–R20 reflects relatively better functioning airways and lung units, whereas N2MBW represents relatively poorly ventilated lung units.^26^ The IOS reveals mechanical properties of the airways reached by the pressure waves, which hypothetically travel preferentially through the more patent of parallel pathways at branch points. In the N2MBW, inert gas slowly clear from poorly ventilated units, causing it to emerge later in the expiration. This significantly increases LCI and indexes derived from phase III slope, that is, Scond and Sacin. Goldman et al. suggest that AX and R5–R20 can be dissociated when bronchoconstriction is the major cause of airflow limitation in the presence of substantially increased bronchomotor tone in proximal airways.^35^ Then R5–R20 is relatively less prominent in comparison with AX. Detected relationship between Scond and AX might suggest that AX provides complementary information about the status of small airways.

In clinical practice, asthma severity is determined mainly on the base of treatment required to maintain asthma control.^1^ In this study, a positive association of GINA score was noted only with LCI within whole peripheral airways measurements. The previous studies on pediatric asthma shown LCI variability from normal value in children with mild disease to slightly elevated in moderately severe disease, what is consistent with this study outcome.^16^, ^38^ This suggests that children with severe asthma are more likely to have an abnormal LCI.^39^

### 4.1 Study limitation

The present study is a cross‐sectional study including a limited number of patients. The results must therefore be interpreted with some caution and the causal relationship cannot be definitely confirmed. However, the main findings are in accordance with recent reports.

Due to lack of reference values of novel IOS parameters, AX and R5–R20, the raw values were used in analysis. There are some studies that have shown that those parameters may be affected by height, age, and gender.^30^ Therefore, the reliability of their relationship with other pulmonary function measurements may be vulnerable to demographic factors. However, included study groups were comparable regarding age, height, weight, and BMI.

Another study limitation is fact that the comparison was done between two groups of patients, and the change of peripheral airway status after recovery from an exacerbation was not assessed.

## 5 CONCLUSIONS

This study provides further evidence of the role of peripheral airways involvement in pediatric asthma reaffirming their clinical relevance to asthma control. The novel finding, which is detection of the acinar zone impairment in most of children with asthma exacerbation, indicates this area of the lung as important additional target for therapy and provides new insights into pathophysiological process of pediatric asthma.

Further studies are needed to clarify what test or combination of tests the best reflects the function of peripheral airways and had the best utility in pediatric asthma.

## AUTHOR CONTRIBUTIONS

**Maria Wawszczak**: study design, patients' enrolment, data acquisition, writing manuscript. **Marek Kulus**: writing manuscript, manuscript critical revision. **Joanna Peradzynska**: study design, data acquisition, data analysis, writing manuscript, manuscript critical revision.

## CONFLICT OF INTEREST

The authors do not declare financial support.

## ETHICS STATEMENT

The authors hereby declares that the study has been reviewed and approved by the Ethics Committee of the Medical University of Warsaw and has been performed in accordance with the ethical standards laid down in the Declaration of Helsinki (version revised in Brazil 2013). The authors hereby confirm that all children and their caregivers gave their informed consent prior to their inclusion in the study.

## Data Availability

The data that support the findings of this study are available on request from the corresponding author. The data are not publicly available due to privacy or ethical restrictions.
